# A Novel Belief Propagation-Based Probabilistic Multiple Hypothesis Tracking Algorithm for Multiple Resolvable Group Targets

**DOI:** 10.3390/e28030273

**Published:** 2026-02-28

**Authors:** Tianli Ma, Peiling Shi, Sai Liu, Peng Wang

**Affiliations:** 1 School of Electronic Information Engineering, Xi’an Technological University, Xi’an 710021, China; matianli111@xatu.edu.cn (T.M.); spl132x@126.com (P.S.); 2 China International Engineering Consulting Corporation, Beijing 100089, China; liusaist@126.com

**Keywords:** multiple group targets, minimum spanning tree, probabilistic multiple hypothesis tracking, belief propagation

## Abstract

A key challenge in multiple group target tracking is to maintain consistent data association in the presence of dynamic evolutions, i.e., splitting and merging. This paper proposes a Belief Propagation-based Multiple Hypothesis Tracking framework. The measurements are partitioned by using the Minimum Spanning Tree divisive clustering algorithm. A factor graph model is then constructed for the association hypotheses between group targets and measurements, followed by the inference of marginal posterior association probabilities via the Belief Propagation. These probabilities are finally integrated into an Expectation-Maximization framework, and the group states are updated by maximizing the expected log-likelihood function. Simulation results demonstrate that the proposed algorithm achieves significantly higher accuracy in the joint estimation of kinematic states and target cardinality compared to the PMHT-based, PHD-based, and JPDA-based algorithms.

## 1. Introduction

The aim of multiple target tracking (MTT) is to simultaneously estimate the time-varying number of targets and their states, based upon imperfect sensor measurement that is typically corrupted by noise and clutters. Multiple target tracking is crucial for many applications, such as pedestrian safety [1], motion and scene analysis [2], and video surveillance [3].

**Figure 1:**
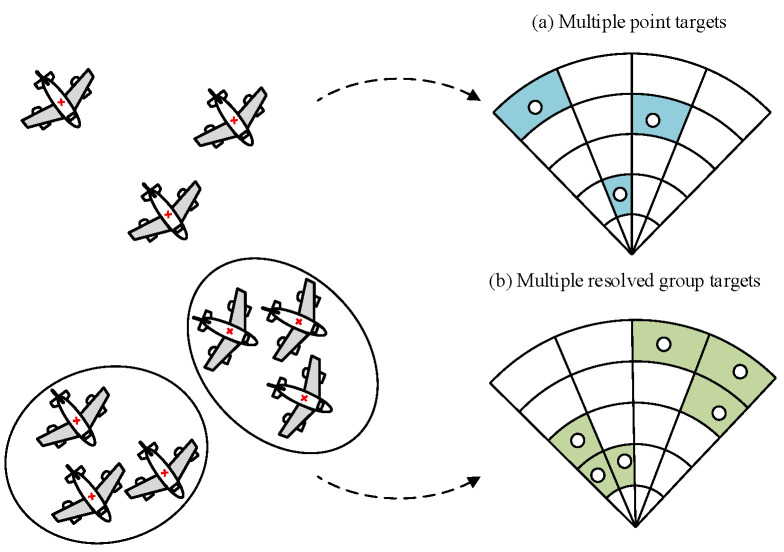
The difference between the multiple point targets and multiple resolvable group targets.

Traditional MTT often makes the assumption that each target can produce at most one measurement at a given time step, and these targets are mutually independent [4]. However, the assumption is unrealistic for group targets, such as drone swarms, bird flocks and crowds, which are composed of multiple resolvable point targets that have certain spatial relationships and the same movement models between individuals. The measurement of each member of the group target is located in different radar resolution units [5,6]. The difference between the multiple point targets and multiple resolvable group targets is shown in Figure 1.

Compared to multiple point target tracking (MPTT), multiple resolvable group target tracking (MRGTT) needs to consider not only the motion state of the group target, but also the structural information.

Similar to the MPTT algorithm, the MRGTT algorithms include the traditional vector model-based algorithms and random finite sets (RFS) approach. The main idea of the first category is to calculate the association probability between the target and measurements. The Joint Probabilistic Data Association (JPDA) [7], the Joint Integrated Probabilistic Data Association filter [8], the Multiple Hypothesis Tracking (MHT) [9], and the Probabilistic MHT (PMHT) may be used [10,11,12]. The main drawback of the above approaches is that the computational complexity grows with the number of measurements. The second category models the targets and measurements using the RFS. It allows multiple-target tracking in the presence of clutter and with uncertain associations to be cast in a Bayesian framework [13], resulting in an optimal multi-target Bayes filter. A Gaussian inverse Wishart probability hypothesis density (GIW-PHD) filter is proposed to handle multiple group targets, where the PHD is approximated using Gaussian inverse Wishart distribution [14]. The cardinality CPHD approach is introduced to MGTT in [15], which estimates the states and cardinality of the group target. PHD and CPHD filters avoid the data association in multi-target tracking, but these algorithms could not explicitly accommodate the estimation of the target trajectory. Hence, an extended target cardinality balanced multi-target multi-Bernoulli filter is proposed [16] that uses the multi-Bernoulli RFS and Poisson-RFS to model the multiple group state and measurements, respectively. It has a better performance in terms of accuracy compared to PHD and CPHD filters.

Splitting and merging are common dynamic behaviors in group target tracking [17]. Specifically, splitting refers to the phenomenon in which an originally associated group target divides into multiple independent subgroups due to external disturbances (e.g., obstacle avoidance). Conversely, merging occurs when multiple independent group targets aggregate into a single group due to motion coherence or task coordination. The key challenge in tracking splitting and merging groups lies in resolving data association under dynamic variations in both the numbers of the groups and subgroups. If a static birth model is adopted, the above-mentioned algorithms may lose track. Hence, Lu et al. [18] proposed a pattern-space-based group target tracking method which maps measurement data sequences from the measurement space to the pattern space via wavelet transform. Subsequently, Kalman filtering is applied in the pattern space before transforming the results back to the measurement space to output corrected measurements. However, the repeated transformations and filtering operations between spaces lead to progressive error accumulation, ultimately degrading tracking accuracy. Recently, Gning, A. et al. proposed a group target tracking algorithm based on evolving graph networks [19]. This method employs a graph-based representation to model group target structures and achieves dynamic structural evolution through joint updates with particle filter samples. However, the requirement for real-time graph construction and updating significantly increases computational complexity.

In this paper, we propose a belief propagation-based probabilistic multiple hypothesis tracking (PMHT-BP) algorithm for multiple resolvable group targets. To reduce data association complexity, the measurement set is first segmented using a minimum spanning tree (MST) divisive clustering algorithm. The group target states are then parameterized by a group division vector. Subsequently, a data association factor graph model is constructed based on group target-measurement hypotheses, and belief propagation (BP) is employed to compute the joint posterior probability density of group target-measurement associations, and the marginal posterior association probability density. Finally, in the expectation-maximization (EM) step, the updated group target states are obtained by maximizing the group target state likelihood function.

The novelty of the proposed algorithm is performance improvement in accuracy compared to the existing algorithms. The main contributions of the work consist of:

1. Group partition vector representation that models the connection relationships of sub-targets within the resolvable group target.
2. We establish a PMHT-BP algorithm to address the multi-group target tracking problem during splitting and merging events. In this framework, the PMHT is employed to generate the potential target-measurement associations. The joint posterior probability density of the group target-measurement and the marginal posterior association probability density are calculated by the BP.
3. We demonstrate performance advantages in simulated scenarios with splitting and merging events.

The rest of paper is organized as follows. The problem formulation and group target models are given in Section 2. The Probabilistic MHT algorithm based on Belief Propagation is given in Section 3. In Section 4, an evaluation of the effectiveness of the proposed method is presented, and conclusions are drawn in Section 5.

## 2. Problem Formulation

Considering that there are $N_{k}$ resolvable group targets and $M_{k}$ measurements at time k, the states of the multiple resolvable group targets and the measurements of a sensor can be respectively modeled by the states set $X_{k}$ and measurements set $Z_{k}$ as follows,

(1) $$\begin{array}{c} X_{k}=\{X_{k,1},X_{k,2},\ldots X_{k,N_{k}}\}\\ Z_{k}=\{z_{k,1},z_{k,2},\ldots z_{k,M_{k}}\} \end{array}$$

where $X_{k,i}$ is the state set of the *i*-th resolvable group target, $X_{k,i}=\{x_{k,i}^{l},x_{k,i}^{1},\ldots x_{k,i}^{n_{i}}\}$. Each resolvable group target consists of a single parent target and multiple child targets. $x_{k,i}^{l}$ and $x_{k,i}^{j}$ represent the state of the parent target and the *j*-th child target of the *i*-th resolvable group target at time k, respectively. The target number of the *i*-th resolvable group target is $n_{i}+1$. $z_{k,i}$ represents the *i*-th measurement received by the sensor at the time k. Note that the measurement set $Z_{k}$ contains the measurement generated by the existing group target, and the birth group target as well as the clutter. Here, the number of clutter measurements generated is a Poisson-distributed random variable with parameter $\lambda_{c}$.

Assume the dynamic and measurement models of the *j*-th child target of the *i*-th resolvable group target are as follows:

(2) $$x_{k,i}^{j}=F_{k-1,i}^{l}x_{k-1,i}^{l}+b_{k-1,i}(l,j)+\omega_{k,i}^{j}$$

(3) $$z_{k,i}^{j}=H_{k}x_{k,i}^{j}+v_{k,i}^{j}$$

where $F_{k-1,i}^{l}$ denotes the state transition matrix of the parent target and $H_{k}$ is the observation matrix. $\omega_{k,i}^{j}$ and $v_{k,i}^{j}$ indicate the process and measurement noise, which are assumed to be Gaussian distributions with covariance matrices $Q_{k,i}^{j}$ and $R_{k,i}^{j}$, respectively. $b_{k-1,i}(l,j)$ represents the displacement vector between the parent target l and child target j, such that

(4) $$b_{k-1,i}(l,j)={\left[D_{k-1}(l,j)\times \cos(\theta_{k-1}(l,j)-\phi_{k-1}(l,j)),0,\;D_{k-1}(l,j)\times \sin(\theta_{k-1}(l,j)-\phi_{k-1}(l,j)),0\right]}^{T}$$

where $D_{k-1}(l,j)$ and $\phi_{k-1}(l,j)$ are the distance and designed angle between the parent target l and child target j, respectively. $\theta_{k-1}(l,j)$ is the motion direction for the parent target l at the time k, $\theta_{k-1}\left(l,j\right)=\arctan({\dot{y}}_{k-1}^{l}/{\dot{x}}_{k-1}^{l})$ [20]. ${\dot{x}}_{k-1}^{l}$ and ${\dot{y}}_{k-1}^{l}$ denote the velocity in the *X*-axis and *Y*-axis, respectively.

Remark: The motions of the child targets are influenced by the parent target, which is not dependent on any target. When l=j, Equation (2) represents the dynamic model of the parent target; otherwise, Equation (2) represents the dynamic model of the child target [21].

For the data association problem of multiple resolvable group target tracking in splitting and merging scenarios, the traditional algorithms perform poorly under the high-density clutter. Hence, a belief propagation-based probabilistic multiple hypothesis tracking (PMHT-BP) algorithm is presented to solve this problem.

## 3. The Proposed Algorithm

### 3.1. The MST-DCA

To effectively handle the measurement uncertainty arising from clutter, the MST-DCA is used to segment the measurements, which partitions the measurement set into distinct clusters and facilitates the subsequent BP-based data association.

The minimum spanning tree divisive clustering algorithm (MST-DCA) is used to exact the potential resolvable group target set. We applied preliminary random partitioning on the measurement set $Z_{k}$, and calculated the group distance $d_{c}$, $d_{c}=\gamma \cdot M_{k}^{-d^{-1}}$, where $d_{c}$ denotes the feature dimension of measurements and γ is a scaling factor. Define the empty set Φ. For each measurement $z_{k,i}$, if the distance is less than or equal to the group radius $d_{c}$, the measurement $z_{k,i}$ is assigned to the existing group $\Phi_{e}$. If no group matches, a new group is created. This process is repeated until all measurements are assigned. The MST-DCA employs a density threshold ε to distinguish between core and boundary groups. A group is identified as a core group $\Phi_{c}$ if its density exceeds $\varepsilon =f([\alpha_{c}\times \alpha_{g}]-1)$, in which $\alpha_{c}$ denotes the proportion of $\Phi_{c}$, $\alpha_{g}$ is the number of the *i*-th group $\Phi_{i}$, and f(i) is the density of the *i*-th group sorted in descending order.

The minimum spanning tree for core groups and pruned weight of the longest edge is established, incorporating boundary group measurements into adjacent clusters if the Euclidean distance to the core groups $\Phi_{c}$ is less than $3\times \alpha_{c}\times d_{c}$. Otherwise, these measurements are retained in the boundary groups $\Phi_{b}$. The core group is the partitioned measurement set ${\tilde{Z}}_{k}$.

### 3.2. Group Partition Vector

**Definition** **1.** *An existing target is defined as a target with state* ${\underline{x}}_{k}$  *at time step* k *that will survive in time step* k+1*. The existing target set is defined as* $PT_{S}$*,* $PT_{S}=\{{\underline{x}}_{k}^{1},{\underline{x}}_{k}^{2},\ldots {\underline{x}}_{k}^{n_{s}}\}$. $n_{s}$ *is the number of the existing target. A binary variable* ${\underline{r}}_{k}^{i}$ *describes whether the target exists at the time* k*. If the target exists, then* ${\underline{r}}_{k}^{i}=1$*; otherwise,* ${\underline{r}}_{k}^{i}=0$.

**Definition** **2.** *A new birth target is defined as a completely new target with state* ${\bar{x}}_{k}$ *detected by the sensor at time step* k*. The new birth target set is defined as* $PT_{B}$*,* $PT_{B}=\{{\bar{x}}_{k}^{1},{\bar{x}}_{k}^{2},\ldots ,{\bar{x}}_{k}^{n_{b}}\}$. $n_{b}$ *is the number of the new birth target. A binary variable* ${\bar{r}}_{k}^{j}$ *describes whether the target exists at the time* k*. If the target exists, then* ${\bar{r}}_{k}^{j}=1$*; otherwise, *${\bar{r}}_{k}^{j}=0$.

**Figure 2:**
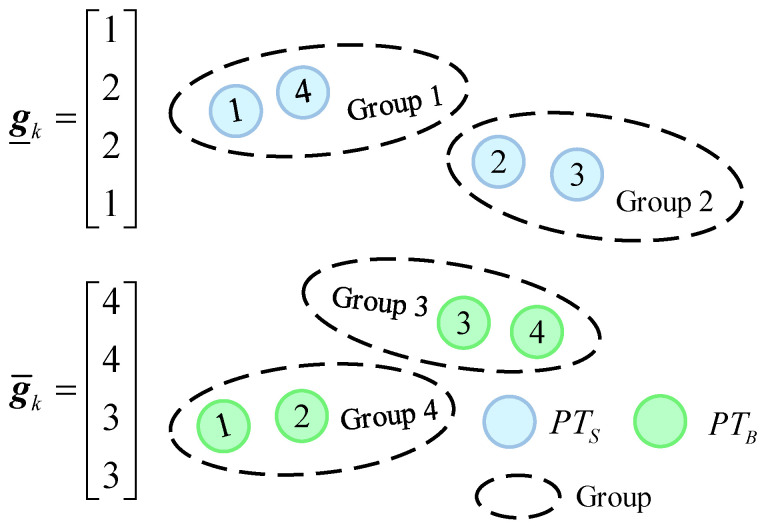
An example of the group partition vectors. The vector ${\underline{g}}_{k}$ shows the assignment of surviving targets to groups, with targets 1 and 4 in Group 1 and targets 2 and 3 in Group 2. Similarly, ${\bar{g}}_{k}$ shows the assignment for new birth targets, with targets 3 and 4 in Group 3 and targets 1 and 2 in Group 4.

The group partition vector $g_{k}$ represents the group structure and constitutes the discrete state space $G_{k}$, $g_{k}=[{\underline{g}}_{k},{\bar{g}}_{k}]\in G_{k}$, where $G_{k}\subset {\left\{1,2,\ldots ,\max(g_{k})\right\}}^{n_{s}+n_{b}}$. ${\underline{g}}_{k}$ and ${\bar{g}}_{k}$ are defined as the structure of the existing target and new birth target, respectively: ${\underline{g}}_{k}={\left[{\underline{g}}_{k}^{1},\ldots ,{\underline{g}}_{k}^{n_{s}}\right]}^{T}$ and ${\bar{g}}_{k}={\left[{\bar{g}}_{k}^{1},\ldots ,{\bar{g}}_{k}^{n_{b}}\right]}^{T}$. An example of the group partition vectors is shown in Figure 2.

Suppose that the joint states of the group targets are defined as $\Pi_{k}=\{{\underline{\pi }}_{k}^{1:n_{s}},{\bar{\pi }}_{k}^{1:n_{b}}\}$. ${\underline{\pi }}_{k}^{1:n_{s}}=\{{\underline{x}}_{k}^{1:n_{s}},{\underline{r}}_{\;k}^{1:n_{s}}\}$ and ${\bar{\pi }}_{k}^{1:n_{b}}=\{{\bar{x}}_{k}^{1:n_{b}},{\bar{r}}_{k}^{1:n_{b}}\}$ represent the states of the existing target and the new birth target. To quantify the previous information of different group partitions at the time k−1, the group partition transition probability $p({\underline{g}}_{k}|x_{k-1},r_{k-1})$ is given by

(5) $$p({\underline{g}}_{k}|x_{k-1},r_{k-1})=\frac{\varphi ({\underline{g}}_{k}|x_{k-1},r_{k-1})}{\sum_{{{\underline{g}}^{\prime }}_{k}\in G_{k}}\varphi ({{\underline{g}}^{\prime }}_{k}|x_{k-1},r_{k-1})}$$

where $\varphi ({\underline{g}}_{k}|x_{k-1},r_{k-1})$ is the evaluation function that measures the state similarity of each target relative to the centroid of its associated group.

(6) $$\varphi ({\underline{g}}_{k}|x_{k-1},r_{k-1})=\prod_{i\in C_{k}}p_{i,g_{k}^{i}}\prod_{j\neq g_{k}^{i}}\left(1-p_{i,j}\right)$$

where $C_{k}=\{i|r_{k-1}^{i}=1\}$ is index set of all targets at time k−1 and $g_{k}^{i}$ is the group label of target i in the group partition vector. $P_{i,j}$ denotes the probability that target i belongs to group j.

### 3.3. The PMHT-BP Algorithm

In this section, a novel PMHT-BP algorithm, which integrates the PMHT framework within the Belief Propagation (BP) algorithm, is proposed. The marginal posterior association probabilities are iteratively approximated to calculate the likelihood function. A Kalman smoother is then employed to find the state sequence that maximizes this likelihood function, obtaining a sequence of estimated group target states under the maximum a posteriori criterion.

The logarithmic form of the maximum likelihood parameter estimation problem to be solved is presented as follows:

(7) $$\begin{array}{c} \Pi^{MAP}=\arg\underset{\Pi_{k}}{\max}\log p(\Pi_{k}|{\tilde{Z}}_{k})\\ \;=\arg\underset{\Pi_{k}}{\max}\log p(\Pi_{k},{\tilde{Z}}_{k}) \end{array}$$

where $p(\Pi_{k}|{\tilde{Z}}_{k})$ is the joint posterior probability density function. As the dimensionality grows exponentially with the number of targets and measurements, direct computation becomes infeasible. To address this intractability, the complete data is introduced. This transforms the original maximum likelihood parameter estimation into an iterative process of solving for the expectation of the complete-data log-likelihood function.

Define the complete association set $\left({\tilde{Z}}_{k},A_{k},B_{k}\right)$; $A_{k}=\{a_{k}^{1},\ldots ,a_{k}^{n_{k}}\}$ and $B_{k}=\{b_{k}^{1},\ldots ,b_{k}^{m_{k}}\}$ are the association sets for the *i*-th target and *j*-th measurement at time k, respectively. When the *i*-th target is associated with the *j*-th measurement, then $a_{k}^{i}\;=\;j$ and $b_{k}^{j}=i$; otherwise, $a_{k}^{i}=0$ and $b_{k}^{j}=0$.

#### 3.3.1. E-Step

Given the complete association set $\left({\tilde{Z}}_{k},A_{k},B_{k}\right)$ at time k, the expectation of the joint state log-likelihood function is expressed as

(8) $$\begin{array}{c} Q(\Pi_{k},\Pi_{k}^{l})=E\{\log p({\tilde{Z}}_{k},A_{k},B_{k}|\Pi_{k})|{\tilde{Z}}_{k},\Pi_{k}^{l}\}\\ \;=\sum_{A_{k},B_{k}}\log p(\Pi_{k},{\tilde{Z}}_{k},A_{k},B_{k})p(A_{k},B_{k}|\Pi_{k}^{l},{\tilde{Z}}_{k}) \end{array}$$

where l is the iteration index. $\Pi_{k}^{l}$ is the estimated state from the l-th iteration. Based on the Bayes theorem, the joint probability of group target states, measurements, and association variables is

(9) $$p(\Pi_{k},{\tilde{Z}}_{k},A_{k},B_{k})=p(\Pi_{0})\prod_{k=1}^{T}p(\Pi_{k}|\Pi_{k-1})\;p({\tilde{Z}}_{k},A_{k},B_{k}|\Pi_{k})$$

where $p(\Pi_{\;0})$ is the initial prior of the group target state. $p(\Pi_{k}|\Pi_{k-1})$ is the state transition probability.

Because the complete association sets $A_{k}$ and $B_{k}$ are independent, $p(A_{k})=\prod_{i}^{n_{k}}p(a_{k}^{i})$ and $p(B_{k})=\prod_{j}^{m_{k}}p(b_{k}^{j})$. Hence, the joint probability can be obtained as follows:

(10) $$p({\tilde{Z}}_{k},A_{k},B_{k}|\Pi_{k})\propto (\prod_{i=1}^{n_{k}}\psi ({\underline{x}}_{k}^{i},{\underline{r}}_{k}^{\;i},a_{k}^{i},z_{k}^{i})\prod_{j=1}^{m_{k}}\psi ({\bar{x}}_{k}^{j},{\bar{r}}_{k}^{j},b_{k}^{j},z_{k}^{j}))\psi (A_{k},B_{k})$$

Under the assumption of mutual independence among association variables, the association probabilities between target and measurement can be calculated by marginalizing

(11) $$p(A_{k},B_{k}|\Pi_{k},{\tilde{Z}}_{k})=\prod_{k=1}^{T}p(A_{k},B_{k}|\Pi_{k},{\tilde{Z}}_{k})$$

with $p(A_{k},B_{k}|\Pi_{k},{\tilde{Z}}_{k})\propto p({\tilde{Z}}_{k},A_{k},B_{k}|\Pi_{k})$.

Substituting Equations (9) and (11) into Equation (8), the auxiliary function to be maximized by

(12) $$\begin{array}{c} Q(\Pi_{k},\Pi_{k}^{l})\\ =\log p(\Pi_{k})+E\{\log[p({\tilde{Z}}_{k},A_{k},B_{k}|\Pi_{k})]|{\tilde{Z}}_{k},\Pi_{k}^{l}\}\\ =\log p(\Pi_{k})+\sum_{A_{k},B_{k}}\log[p({\tilde{Z}}_{k},A_{k},B_{k}|\Pi_{k})]\;p(A_{k},B_{k}|{\tilde{Z}}_{k},\Pi_{k}^{l}) \end{array}$$

with

(13) $$\log p\left(\Pi_{k}\right)=\log[p\left(\Pi_{0}\right)\prod_{k=1}^{T}p\left(\Pi_{k}|\Pi_{k-1}\right)]$$

Then, arranging Equations (12) and (13) as follows,

(14) $$\begin{array}{c} Q(\Pi_{k},\Pi_{k}^{l})=\log p\left(\Pi_{0}\right)+\sum_{k=1}^{T}\log p\left(\Pi_{k}|\Pi_{k-1}\right)\\ \;+\sum_{k=1}^{T}\sum_{A_{k},B_{k}}\left\{\log p({\tilde{Z}}_{k},A_{k},B_{k}|\Pi_{k})\}p(A_{k},B_{k}|{\tilde{Z}}_{k},\Pi_{k}^{l}\right) \end{array}$$

Substituting Equation (10) into Equation (14), the expression can be simplified as

(15) $$\begin{array}{c} Q(\Pi_{k},\Pi_{k}^{l})=\log p\left(\Pi_{0}\right)+\sum_{k=1}^{T}\log p\left(\Pi_{k}|\Pi_{k-1}\right)\\ \;+\sum_{k=1}^{T}(\sum_{i=1}^{n_{k}}\sum_{a_{k}^{i}=0}^{m_{k}}\omega_{k}^{i}(a_{k}^{i})\log\psi ({\underline{x}}_{k}^{i},1,a_{k}^{i},z_{k}^{i})\\ \;+\sum_{j=1}^{m_{k}}\sum_{b_{k}^{j}=0}^{n_{k}}\omega_{k}^{j}(b_{k}^{j})\log\psi ({\bar{x}}_{k}^{j},1,b_{k}^{j},z_{k}^{j})) \end{array}$$

where $\omega_{k}^{i}(a_{k}^{i})=p(a_{k}^{i}|{\underline{\pi }}_{k}^{i},{\tilde{Z}}_{k})$ and $\omega_{k}^{j}(b_{k}^{j})=p(b_{k}^{j}|{\bar{\pi }}_{k}^{j},{\tilde{Z}}_{k})$ are the marginal posterior association probabilities, respectively.

Given the measurement set ${\tilde{Z}}_{k}$, the association probability of the *i*-th target $\psi ({\underline{x}}_{k}^{i},1,a_{k}^{i},z_{k}^{i})$ and association probability of the *j*-th measurement $\psi ({\bar{x}}_{k}^{j},1,b_{k}^{j},z_{k}^{j})$ at the time k are given by

(16) $$\psi ({\underline{x}}_{k}^{i},1,a_{k}^{i},z_{k}^{i})=\left\{\begin{array}{ll} \frac{P_{d}({\underline{x}}_{k}^{i})p(z_{k}^{a_{k}^{i}}|{\underline{x}}_{k}^{i})}{\lambda_{\mathrm{fa}}(z_{k}^{a_{k}^{i}})}, & a_{k}^{i}\neq 0\\ 1-P_{d}({\underline{x}}_{k}^{i}), & a_{k}^{i}=0. \end{array}\right.$$

(17) $$\psi ({\bar{x}}_{k}^{j},1,b_{k}^{j},z_{k}^{j})=\left\{\begin{array}{ll} 0, & b_{k}^{j}\neq 0\\ \frac{P_{d}({\bar{x}}_{k}^{j})p(z_{k}^{b_{k}^{j}}|{\bar{x}}_{k}^{j})}{\lambda_{\mathrm{fa}}(z_{k}^{b_{k}^{j}})}, & b_{k}^{j}=0. \end{array}\right.$$

Based on the consistency constraint [22], we obtain the consistency factor

(18) $$\psi (A_{k},B_{k})=\left\{\begin{array}{l} 0,\;a_{k}^{i}=j,b_{k}^{j}\neq i\;or\;a_{k}^{i}\neq j,b_{k}^{j}=i\\ 1,\;other \end{array}\right.$$

#### 3.3.2. Factor Graphs Model

According to the group partition vector and the independence assumption, the joint probability density function $p(\Pi_{k},G_{k},A_{k},B_{k}|{\tilde{Z}}_{k})$ is given as follows:

(19) $$\begin{array}{l} p(\Pi_{k},G_{k},A_{k},B_{k}|{\tilde{Z}}_{k})\propto p({\tilde{Z}}_{k},A_{k},B_{k},\Pi_{k},G_{k})\\ =\prod_{k=1}^{T}p(z_{k},a_{k},b_{k},\pi_{k},g_{k}|\pi_{k-1},g_{k-1})\\ =\prod_{k=1}^{T}p(z_{k},a_{k},b_{k},{\bar{\pi }}_{k},{\bar{g}}_{k}|{\underline{\pi }}_{k},{\underline{g}}_{k})p({\underline{\pi }}_{k},{\underline{g}}_{k}|\pi_{k-1},g_{k-1}) \end{array}$$

where $G_{k}=\left\{g_{1},g_{2},\ldots ,g_{k}\right\}$ is the sequence of group partition vectors up to the time step k. The transition probability density function of the extended state and group partition vector $p({\underline{\pi }}_{k},{\underline{g}}_{k}|\pi_{k-1},g_{k-1})$ is defined as

(20) $$p({\underline{\pi }}_{k},{\underline{g}}_{k}|\pi_{k-1},g_{k-1})=p({\underline{\pi }}_{k}|{\underline{g}}_{k},\pi_{k-1},g_{k-1})p({\underline{g}}_{k}|\pi_{k-1},g_{k-1})$$

Since the measurements and group partition vector are irrelevant, the conditional probability density function is as follows:

(21) $$\begin{array}{l} p(z_{k},a_{k},b_{k},{\bar{\pi }}_{k},{\bar{g}}_{k}|\;{\underline{\pi }}_{k},{\underline{g}}_{k})\\ =p(z_{k},a_{k},b_{k},{\bar{x}}_{k},{\bar{r}}_{k},{\bar{g}}_{k}|\;{\underline{x}}_{k},{\underline{r}}_{k},{\underline{g}}_{k})\\ =p(z_{k}|\;a_{k},b_{k},{\bar{x}}_{k},{\bar{r}}_{k},{\bar{g}}_{k},{\underline{x}}_{k},{\underline{r}}_{k},{\underline{g}}_{k}) \end{array}$$

(22) $$\begin{array}{l} p(a_{k},b_{k},{\bar{x}}_{k},{\bar{r}}_{k},{\bar{g}}_{k}|\;{\underline{x}}_{k},{\underline{r}}_{k},{\underline{g}}_{k})\\ =p(z_{k}|\;a_{k},b_{k},{\bar{x}}_{k},{\bar{r}}_{k},{\underline{x}}_{k},{\underline{r}}_{k})p(a_{k},b_{k},{\bar{x}}_{k},{\bar{r}}_{k}|\;{\underline{x}}_{k},{\underline{r}}_{k}) \end{array}$$

Assuming that the new birth target and the existing target are independent of each other at the time k, we obtain

(23) $$\begin{array}{l} p(a_{k},b_{k},{\bar{x}}_{k},{\bar{r}}_{k}|\;{\underline{x}}_{k},{\underline{r}}_{k})\\ =p({\bar{x}}_{k}|\;a_{k},b_{k},{\bar{r}}_{k},{\underline{x}}_{k},{\underline{r}}_{k})p(a_{k},b_{k},{\bar{r}}_{k}|\;{\underline{x}}_{k},{\underline{r}}_{k})\\ =p({\bar{x}}_{k}|\;{\bar{r}}_{k})p(a_{k},b_{k},{\bar{r}}_{k}|\;{\underline{x}}_{k},{\underline{r}}_{k}) \end{array}$$

Substituting Equations (16)–(18) in Equation (21), when $r_{k}=1$, we obtain

(24) $$\begin{array}{l} p(z_{k},a_{k},b_{k},{\bar{\pi }}_{k},{\bar{g}}_{k}|\;{\underline{\pi }}_{k},{\underline{g}}_{k})\\ \propto \psi (a_{k},b_{k})\prod_{i=1}^{n_{k}}\psi ({\underline{x}}_{k}^{i},{\underline{r}}_{k}^{i},a_{k}^{i},z_{k}^{i})\prod_{j=1}^{m_{k}}\psi ({\bar{x}}_{k}^{j},{\bar{r}}_{k}^{j},a_{k},b_{k})\;\psi ({\bar{x}}_{k}^{j},{\bar{r}}_{k}^{j},b_{k}^{j},z_{k}^{j}) \end{array}$$

Substituting Equations (20) and (24) into Equation (19), the joint posterior probability density function is given by

(25) $$\begin{array}{l} p(\Pi_{k},G_{k},A_{k},B_{k}|\;{\tilde{Z}}_{k})\\ =p({\underline{\pi }}_{0})\prod_{k=1}^{T}\left(p({\underline{\pi }}_{k},{\underline{g}}_{k}|\pi_{k-1},g_{k-1}\right)\;\\ \prod_{i=1}^{n_{k}}\psi ({\underline{x}}_{k}^{i},{\underline{r}}_{k}^{i},a_{k}^{i},z_{k}^{i})\psi (a_{k},b_{k})\prod_{j=1}^{m_{k}}\psi ({\bar{x}}_{k}^{j},{\bar{r}}_{k}^{j},b_{k}^{j},z_{k}^{j})) \end{array}$$

**Figure 3:**
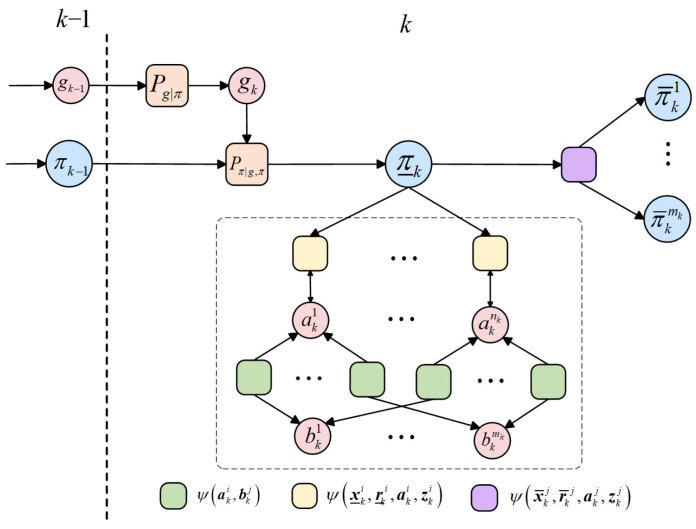
The factor graph description.

The factorization structure Equation (25) can be represented by the factor graph model. In a factor graph, each parameter variable is represented by a variable node and each factor by a factor node (depicted in Figure 3 by a circle and a square, respectively). Variable node and factor node are adjacent, i.e., connected by an edge, if the variable is an argument of the factor.

#### 3.3.3. M-Step

Our aim in this section is to find the MAP state estimate of $\Pi_{k}$ in Equation (15):

(26) $$\Pi^{MAP}=\arg\underset{\Pi_{k}}{\max}Q(\Pi_{k},\Pi_{k}^{l})$$

Let $\Pi_{k}^{l}=\Pi^{MAP}$ and rewrite Equation (14) as follows:

(27) $$\begin{array}{c} \exp(Q(\Pi_{k},\Pi_{k}^{l}))\\ \propto p(\Pi_{0})\prod_{k=1}^{T}p(\Pi_{k}|\;\Pi_{k-1})\sum_{b_{k}^{j}=0}^{n_{k}}\omega_{k}^{j}(b_{k}^{j})\log\psi ({\bar{x}}_{k}^{j},1,b_{k}^{j},z_{k}^{j}))\\ \prod_{k=1}^{T}\prod_{i=1}^{n_{k}}\prod_{j=1}^{m_{k}}\exp(\sum_{a_{k}^{i}=0}^{m_{k}}\omega_{k}^{i}(a_{k}^{i})\log\psi ({\underline{x}}_{k}^{i},1,a_{k}^{i},z_{k}^{i}) \end{array}$$

The M-step determines the updated estimate $\Pi_{k}^{l+1}$ that maximizes the Q-function defined in Equation (27). This maximization is performed efficiently by a Kalman smoother, which requires the synthetic measurements ${\bar{z}}_{k}^{i}$ and covariances ${\bar{R}}_{k}^{i}$ as follows:

(28) $${\bar{z}}_{k}^{i}=\frac{\sum_{a_{k}^{i}=1}^{m_{k}}\omega_{k}^{i}(a_{k}^{i})z_{k}^{a_{k}^{i}}}{\sum_{a_{k}^{i}=1}^{m_{k}}\omega_{k}^{i}(a_{k}^{i})}$$

(29) $${\bar{R}}_{k}^{i}=\frac{R_{k}^{i}}{\sum_{a_{k}^{i}=1}^{m_{k}}\omega_{k}^{i}(a_{k}^{i})}$$

The proposed algorithm iterates until the following convergence is satisfied:

(30) $$\left\|\Pi_{k}^{l+1}-\Pi_{k}^{l}\right\|<10^{-4}$$

### 3.4. The PMHT-BP Implementation

In this section, an implementation of the PMHT-BP filter is presented for the prediction of existing targets and new birth targets according to Definitions 1 and 2, respectively. The probability $\beta_{k}({\underline{g}}_{k})$ passed from factor node $p_{\underline{g}|\;\pi }$ to the variable nodes corresponding to the feature-oriented variables ${\underline{g}}_{k}$ are calculated as

(31) $$\beta_{k}({\underline{g}}_{k})=\sum_{r_{k-1}\in \{0,1\}}\int p({\underline{g}}_{k}|\;x_{k-1},r_{k-1})p(x_{k-1},r_{k-1})dx_{k-1}$$

where the beliefs $p(x_{k-1},r_{k-1})$ are approximations of the respective marginal posterior probability density function $p(x_{k-1},r_{k-1}|\;z_{1:k-1})$:

(32) $$p(x_{k-1},r_{k-1}|\;z_{1:k-1})=\prod_{i=1}^{n_{k-1}}p(x_{k-1}^{i}|\;z_{1:k-1})p(r_{k-1}^{i}|\;z_{1:k-1})$$

with $p(r_{k-1}^{i}|\;z_{1:k-1})\approx 1-\omega_{k}^{i}$. The messages $\beta_{k}({\underline{\pi }}_{k}|\;{\underline{g}}_{k})$ passed from factor node $p_{\underline{\pi }|\;\underline{g},\pi }$ to the variable nodes corresponding to the feature-oriented variables ${\underline{\pi }}_{k}$ are calculated as

(33) $$\beta_{k}({\underline{\pi }}_{k}|\;{\underline{g}}_{k})=\sum_{r_{k-1}\in \{0,1\}}\int p(x_{k-1},r_{k-1})p({\underline{x}}_{k},{\underline{r}}_{k}|\;{\underline{g}}_{k},x_{k-1},r_{k-1})dx_{k-1}$$

with the initialization at k=1 (i.e., $p({\underline{x}}_{1},{\underline{r}}_{\;1}|\;g_{1},{\underline{x}}_{0},{\underline{r}}_{\;0})=1$); the state transition probability for a surviving target when k>1 (where $r_{k}=1$ and $r_{k-1}=1$) is modeled as

(34) $$p({\underline{x}}_{k},1|\;g_{k},x_{k-1},1)=p_{s}\cdot p({\underline{x}}_{k}|\;x_{k-1},{\underline{g}}_{k})$$

where $p_{s}$ is existence probability.

1. Measurement Evaluation: After the prediction step, messages $\eta_{k}(a_{k}^{i})$ and $\kappa (b_{k}^{j})$ are calculated as
  (35) $$\eta_{k}(a_{k}^{i})=\int \psi ({\underline{x}}_{k}^{i},1,a_{k}^{i},z_{k}^{i})p({\underline{x}}_{k},1)d{\underline{x}}_{k}+\left|M_{a}\right|\int p({\underline{x}}_{k},0)d{\underline{x}}_{k}$$
  (36) $$\kappa (b_{k}^{j})=\int \psi ({\bar{x}}_{k}^{j},1,b_{k}^{j},z_{k}^{j})d{\bar{x}}_{k}+1$$
2. Data Association: In the data association step, by using $\eta_{k}(a_{k}^{i})$, the messages $\xi_{j\to i}^{l}(a_{k}^{i})$ and $\zeta_{i\to j}^{l}(b_{k}^{j})$ are calculated by the loopy BP:

(37) $$\xi_{j\to i}^{l}(a_{k}^{i})=\sum_{b_{k}^{j}=0}^{n_{k}}\kappa (b_{k}^{j})\psi (a_{k}^{i},b_{k}^{j})\prod_{\begin{array}{c} i\in \{1,\ldots ,n_{k}\}\\ \backslash \{i\} \end{array}}\zeta_{i\prime \to j}^{l-1}(b_{k}^{j})$$

(38) $$\zeta_{i\to j}^{l}(b_{k}^{j})=\sum_{a_{k}^{i}=0}^{m_{k}}\eta_{k}(a_{k}^{i})\psi (a_{k}^{i},b_{k}^{j})\prod_{\begin{array}{c} j\in \{1,\ldots ,m_{k}\}\\ \backslash \{j\} \end{array}}\xi_{j\prime \to i}^{l}(a_{k}^{i})$$

and for l=0, Equation (38) is calculated as follows:


(39) $$\zeta_{i\to j}^{0}(b_{k}^{j})=\sum_{a_{k}^{i}=0}^{m_{k}}\eta_{k}(a_{k}^{i})\psi (a_{k}^{i},b_{k}^{j})$$

After the last iteration $l_{end}$, the messages $\xi_{j\to i}^{l_{end}}(a_{k}^{i})$ and $\zeta_{i\to j}^{l_{end}}(b_{k}^{j})$ are multiplied, respectively, i.e.,

(40) $$\nu (a_{k}^{i})=\prod_{j=1}^{m_{k}}\xi_{j\to i}^{l_{end}}(a_{k}^{i})$$

(41) $$\mu (b_{k}^{j})=\prod_{i=1}^{n_{k}}\zeta_{i\to j}^{l_{end}}(b_{k}^{j})$$

3. Measurement Update: The confidence function $p({\underline{x}}_{k}^{i},{\underline{r}}_{k}^{i})$ is calculated as follows, for ${\underline{r}}_{k}^{i}=1$
  (42) $$p({\underline{x}}_{k}^{i},1)=\sum_{a_{k}^{i}=0}^{m_{k}}\psi ({\underline{x}}_{k}^{i},1,a_{k}^{i},z_{k}^{i})\nu (a_{k}^{i})$$
  and for ${\underline{r}}_{k}^{i}=0$
  (43) $$p({\underline{x}}_{k}^{i},0)=\nu (a_{k}^{i}=0)$$
4. Group Partition Vector: Based on the group partition vector, the confidence function is represented by

(44) $$p({\underline{g}}_{k})=\frac{1}{C}\beta_{k}({\underline{g}}_{k})\sum_{r_{k}\in \{0,1\}}\int \beta_{k}({\underline{\pi }}_{k}|\;{\underline{g}}_{k})\prod_{i=1}^{n_{k}}p({\underline{x}}_{k}^{i},{\underline{r}}_{\;k}^{i})d{\underline{x}}_{k}$$

where C is the normalization constant.

5. Marginal Posterior Association Probability: The low-dimensional forms of $\omega_{k}^{i}(a_{k}^{i})$ and $\omega_{k}^{j}(b_{k}^{j})$ in Equation (15) through BP can be obtained:

(45) $$\omega_{k}^{i}(a_{k}^{i})=p(a_{k}^{i}|\;{\tilde{Z}}_{k})=\frac{\nu (a_{k}^{i})}{\sum_{j=0}^{m_{k}}\nu (j)}$$

(46) $$\omega_{k}^{j}(b_{k}^{j})=p(b_{k}^{j}|\;{\tilde{Z}}_{k})=\frac{\mu (b_{k}^{j})}{\sum_{i=0}^{n_{k}}\mu (i)}$$

The pseudo-code of PMHT-BP for a single run is shown in Algorithm 1.
**Algorithm 1:** The pseudo-code of PMHT-BP for a single run**Input:** the measurement set $Z_{k}$, the existing target state ${\underline{\pi }}_{k-1}$, the group partition vector ${\underline{g}}_{k-1}$, the existence probability $p_{s}$, the clutter measurement parameter $\lambda_{c}$, the iteration convergence number of EM algorithm l+1, the iteration convergence number of BP algorithm $l_{end}$
$\mathrm{Output}:\;\mathrm{return}\;x_{k}$
$\;\mathrm{and}\;g_{k}$
1: for k=1 to T **do** $2:\;\mathrm{Partition}\;Z_{k}$$\;\mathrm{into}\;{\tilde{Z}}_{k}$ using MST-DCA; $3:\;\mathrm{Compute}\;\mathrm{prior}\;\mathrm{probability}\;\mathrm{of}\;\mathrm{group}\;\mathrm{partition}\;\beta_{k}({\underline{g}}_{k})$: combining Equations (5), (31) and (32); $4:\;\mathrm{Compute}\;\mathrm{prior}\;\mathrm{probability}\;\mathrm{of}\;\mathrm{existing}\;\mathrm{target}\;\mathrm{states}\;\beta_{k}({\underline{\pi }}_{k}|{\underline{g}}_{k})$: according to Equations (33) and (34); $5:\;\mathrm{while}\;\left\|\Pi_{k}^{l}-\Pi_{k}^{l-1}\right\|>10^{-4}$ **do** $6:\;\mathrm{Initialize}\;\mathrm{messages}\;\eta_{k}(a_{k}^{i})$$,\;\kappa (b_{k}^{j})$: based on Equations (35) and (36); $7:\;\mathrm{while}\;iter<l_{end}$ and Δμ>ε **do**8:                            Update messages: according to Equations (37)–(39);9:                            Compute message difference: $\Delta \mu =\left|\mu_{new}-\mu_{old}\right|$;10:                                      iter←iter+1;11:                  **end while**$12:\;\mathrm{Receive}\;\mathrm{marginal}\;\mathrm{beliefs}\;\nu (a_{k}^{i})$$\;\mathrm{and}\;\mu (b_{k}^{j})$: based on Equations (40) and (41);$13:\;\mathrm{Normalized}\;\mathrm{marginal}\;\mathrm{posterior}\;\mathrm{probability}\;\omega_{k}^{i}(a_{k}^{i})$$\;\mathrm{and}\;\omega_{k}^{j}(b_{k}^{j})$: according to Equations (45) and (46);$14:\;\mathrm{Calculate}\;\mathrm{the}\;\mathrm{synthetic}\;\mathrm{measurements}\;{\bar{z}}_{k}^{i}$$\;\mathrm{and}\;\mathrm{covariances}\;{\bar{R}}_{k}^{i}$: using Equations (28) and (29); $15:\;\mathrm{Update}\;\mathrm{target}\;\mathrm{states}\;x_{k}$$\;\mathrm{within}\;\Pi_{k}^{l+1}$ by maximizing the Q-function; 16:                   l←l+1; 17:       **end while** $18:\;\mathrm{Update}\;\mathrm{group}\;\mathrm{partition}\;\mathrm{vector}\;g_{k}$: through Equation (44);$19:\;\mathrm{Extract}\;\mathrm{target}\;\mathrm{states}\;x_{k}$$\;\mathrm{from}\;\mathrm{the}\;\mathrm{existing}\;\mathrm{targets}\;\mathrm{in}\;\Pi_{k}$; 20: **end for**

### 3.5. Time Complexity

The proposed algorithm is a belief-propagation-based multiple resolvable group target process. The time complexity of the algorithm is mainly composed of two parts, which are the time complexity of the minimum spanning tree divisive clustering algorithm and the belief-propagation-based probabilistic multiple hypothesis tracking algorithm. The time complexity of the minimum spanning tree divisive clustering algorithm is $O(m_{k}^{2})+O(m_{k})$. $O(m_{k}^{2})$ and $O(m_{k})$ are the time complexity of calculating Euclidean distance d and divisive clustering density threshold δ, respectively. $m_{k}$ is the number of measurements. The time complexity of the belief-propagation-based probabilistic multiple hypothesis tracking algorithm is $O((l+1)\cdot l_{end}\cdot n_{k}\cdot m_{k})$. l+1 and $l_{end}$ are the iteration convergence numbers of the EM algorithm and BP algorithm, respectively. $n_{k}$ is the number of targets. The total time complexity of the proposed algorithm is $O(m_{k}^{2}+(l+1)\cdot l_{end}\cdot n_{k}\cdot m_{k})$. The PHD-based, PMHT-based and JPDA-based algorithms are recognized as three effective methods to solve the multiple resolvable group target problem. The time complexities of these algorithms are $O(n_{k}\cdot m_{k})$, $O((l+1)\cdot n_{k}\cdot m_{k})$ and $O(m_{k}^{n_{k}})$, respectively. The complexity of these algorithms are notably related to the value of $m_{k}$ and $n_{k}$. For the multiple resolvable group target system, $n_{k}$ is large and the time complexity of the proposed algorithm is far less than that of the above-mentioned algorithms.

## 4. Simulation Results and Analysis

In this section, the proposed algorithm is tested in diverse tracking scenarios. The optimal sub-pattern assignment (OSPA) metric is used for performance evaluation [16], alongside the F-measure for clustering accuracy assessment [23]. The experiments have been performed on a computer with an Intel G840 2.8 GHz CPU and 4 GB of memory.

### 4.1. Clustering Accuracy Evaluation

In this section, the external quality measure (F-measure) is used to evaluate the clustering quality of the MST-DCA, which is designed as an overall assessment performance that combines the precision and recall ideas from information retrieval [24].

**Figure 4:**
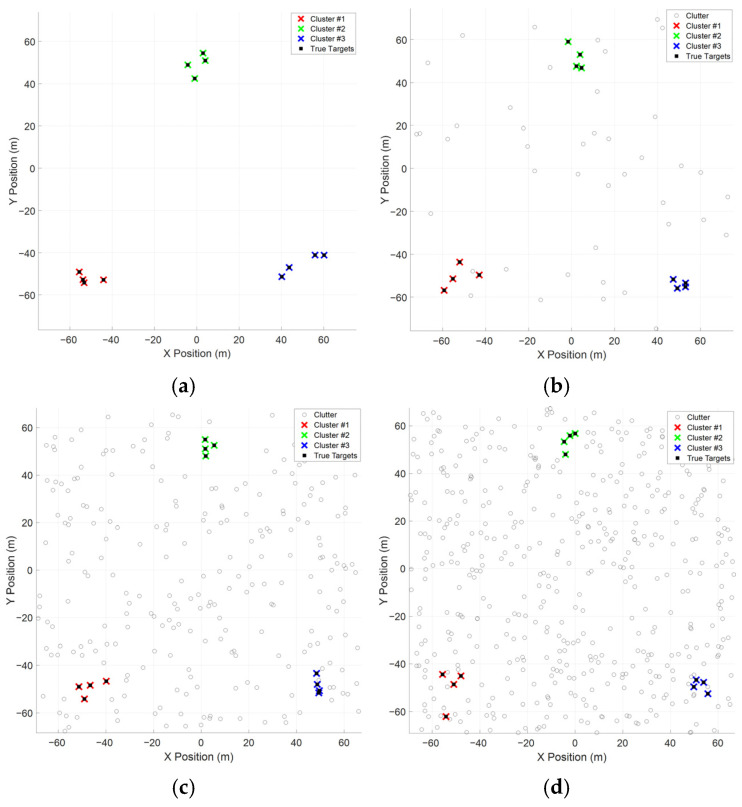
Comparison of classification results. (**a**) Original data set. (**b**) $\lambda_{c}=100$. (**c**) $\lambda_{c}=500$. (**d**) $\lambda_{c}=1000$. The gray circles and black squares represent the clutter and the true targets, respectively. The red, green and blue crosses indicate different clusters classified by the MST-DCA.

**Figure 5:**
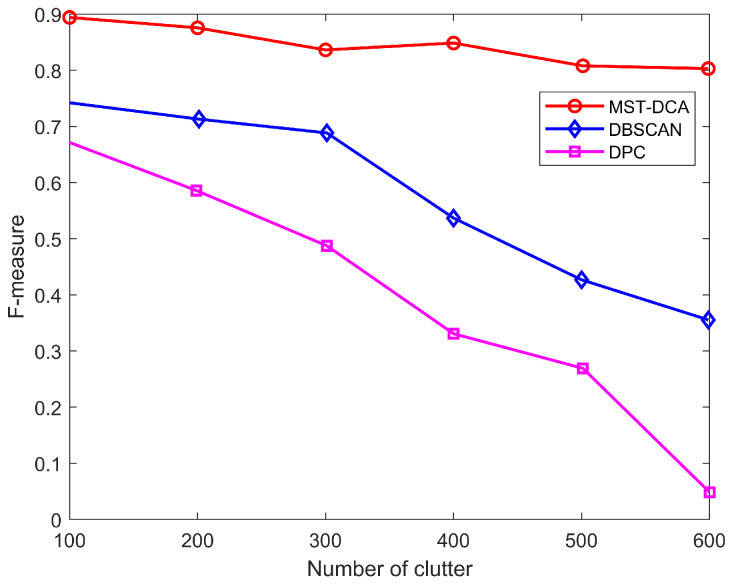
Comparison of clustering algorithm results.

Figure 4 shows the comparison of classification results with different clutter numbers. It is clear that the MST-DCA precisely extract the true group target. In Figure 5, the F-measure with different clutter numbers is carried out in order to compare the performance of the MST-DCA, DBSCAN [25] and DPC [26]. The result illustrates that the DPC and DBSCAN algorithms significantly decrease with the clutter number increase. The MST-DCA is more robust than the DBSCAN and DPC algorithm, because the edge pruning of the MST-DCA dynamically adjusts the cluster boundaries.

### 4.2. Performance Analysis

In this section, we consider the three resolvable group targets that are tracked in a [−500 m,500 m]×[−500 m,500 m] 2D surveillance area. The dynamics of the parent target centroid of the *i*-th group target are described as

(47) $$x_{k,i}^{l}=F_{k-1,i}^{l}x_{k-1,i}^{l}+\omega_{k,i}^{l}$$

where $x_{k,i}^{l}={\left(x_{k,i}^{l},{\dot{x}}_{k,i}^{l},y_{k,i}^{l},{\dot{y}}_{k,i}^{l}\right)}^{T}$ is the state variable, $x_{k,i}^{l},{\dot{x}}_{k,i}^{l}$ are the position and velocity in the *X*-axis, and $y_{k,i}^{l},{\dot{y}}_{k,i}^{l}$ denotes the position and velocity in the *Y*-axis. $F_{k-1,i}^{l}$ denotes the state transition matrix of the parent target of the *i*-th group target at time k−1. The process noise $\omega_{k,i}^{l}$ is the zero-mean Gaussian random vector with covariance matrix $Q_{k,i}^{l}=diag\{0.5,0.1,0.5,0.1\}$. The parent target motion can be modeled by a combination of constant turn (CT) motion and constant velocity (CV) motion. Under the assumption of CV motion, it is defined as follows:

(48) $$F_{k-1,i}^{l}=\left[\begin{array}{cccc} 1 & T & 0 & 0\\ 0 & 1 & 0 & 0\\ 0 & 0 & 1 & T\\ 0 & 0 & 0 & 1 \end{array}\right]$$

where T denotes the sampling time. While in CT motion, it is defined as

(49) $$F_{k-1,i}^{l}=\left[\begin{array}{cccc} 1 & \frac{\sin(\vartheta_{k-1,i}^{l}T)}{\vartheta_{k-1,i}^{l}} & 0 & \frac{\cos(\vartheta_{k-1,i}^{l}T)-1}{\vartheta_{k-1,i}^{l}}\\ 0 & \cos(\vartheta_{k-1,i}^{l}T) & 0 & -\sin(\vartheta_{k-1,i}^{l}T)\\ 0 & \frac{1-\cos(\vartheta_{k-1,i}^{l}T)}{\vartheta_{k-1,i}^{l}} & 1 & \frac{\sin(\vartheta_{k-1,i}^{l}T)}{\vartheta_{k-1,i}^{l}}\\ 0 & \sin(\vartheta_{k-1,i}^{l}T) & 0 & \cos(\vartheta_{k-1,i}^{l}T) \end{array}\right]$$

where $\vartheta_{k-1,i}^{l}$ denotes the turn rate.

The measurements are obtained from radar located in [0m,0m]. The measurement model is given by Equation (3), in which $z_{k,i}^{j}$ is the measurement variable, $z_{k,i}^{j}={\left[x_{k,i}^{j},y_{k,i}^{j}\right]}^{T}$. $H_{k}=\left[\begin{array}{cccc} 1 & 0 & 0 & 0\\ 0 & 0 & 1 & 0 \end{array}\right]$ is the measurement matrix. The measurement noise $v_{k,i}^{j}$ is the zero-mean Gaussian random vector with covariance matrix $R_{k,i}^{j}=I_{2}\sigma_{m}^{2}$, and $\sigma_{m}=10m$. The scenario has 175 time-steps. The clutters are assumed to be Poisson-distributed with mean $\lambda_{c}=10$, respectively. In the scenario with three group targets, the initial state, the appearance and disappearance times, and the merge and split times are shown in Table 1.

**Figure 6:**
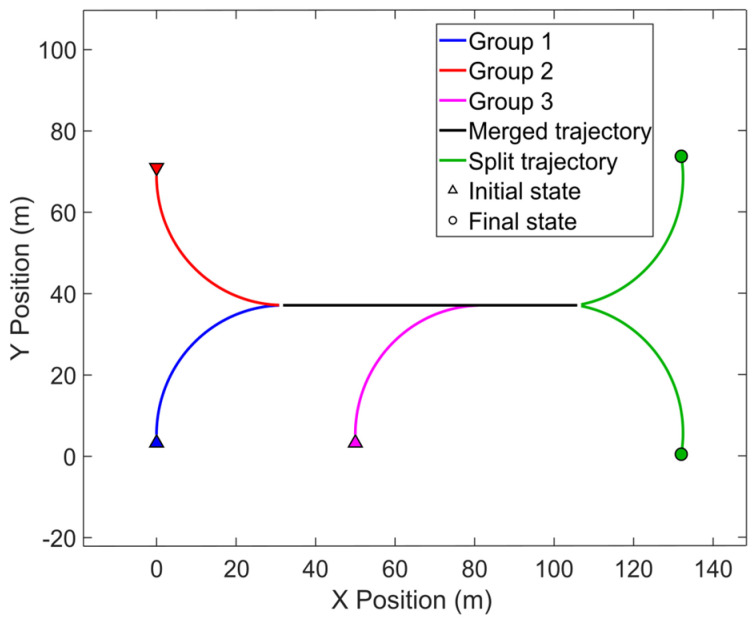
MRGTT merging and splitting trajectories.

**Figure 7:**
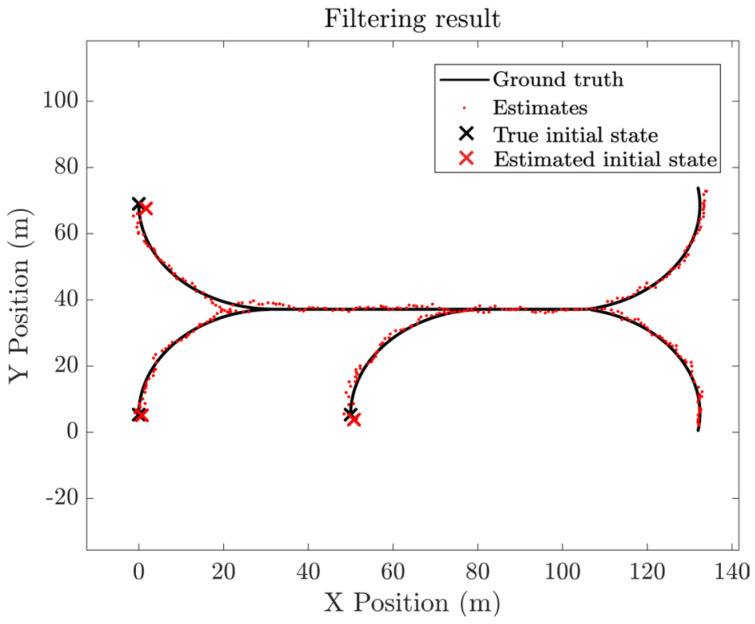
The results of data association of a single run.

Figure 6 depicts the true group target trajectories. The results of data association of a single run are shown in Figure 7. It can be seen that the proposed algorithm has relatively higher tracking accuracy.

To evaluate the performance of the proposed filter against the JPDA-based [7], PMHT-based [12], and PHD-based [14] algorithms, we performed 100 Monte Carlo runs and obtain the optimal sub-pattern assignment (OSPA) error [16] at each time step for each algorithm. The OSPA distance between two random finite sets X and Y is defined as follows:

(50) $$d_{p}^{\left(c\right)}(X,Y)=(\frac{1}{n}(\underset{\pi \in \prod_{n}}{\min}\sum_{i=1}^{m}d^{\left(c\right)}{\left(x_{i},y_{\pi (i)}\right)}^{p}+c^{p}{\left(n-m))\right)}^{1/p}$$

where the function $d_{p}^{\left(c\right)}$ is the OSPA metric of order p with cut-off c, set as p = 2 and c = 8. $n,m\in Ν=\left\{1,2\ldots \right\}$ and $\prod_{n}$ are the set of permutations.

**Figure 8:**
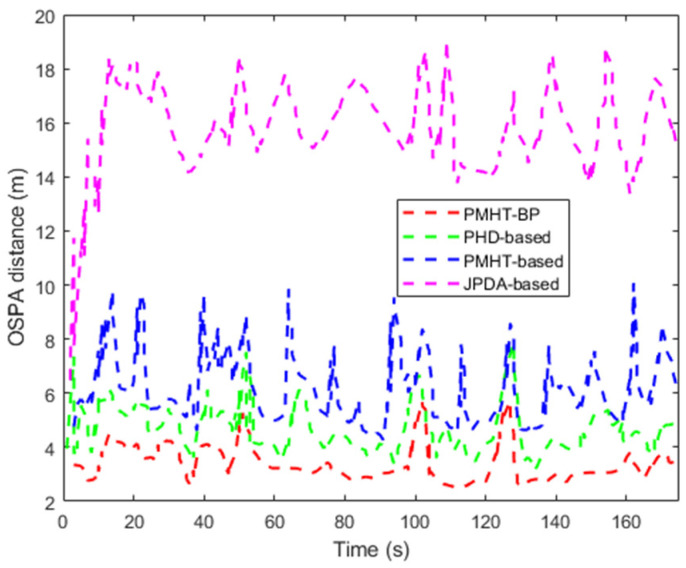
The OSPA distance of position estimates.

**Figure 9:**
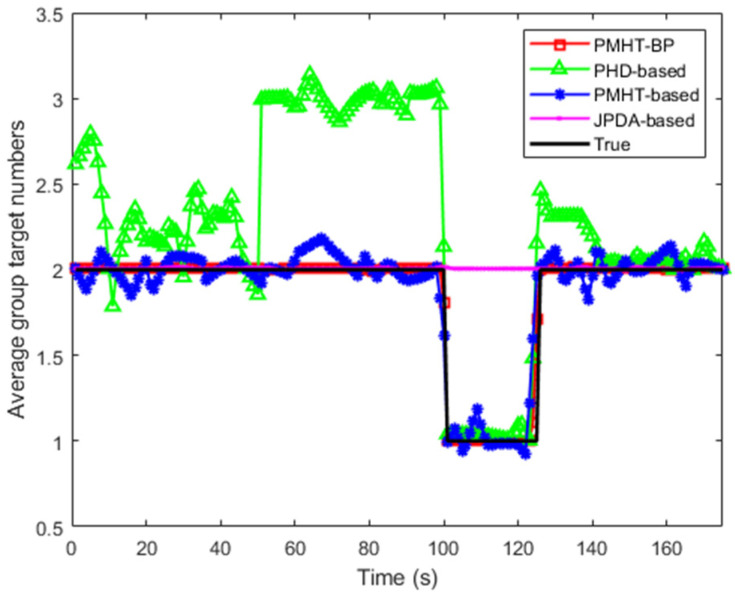
The average of the estimated numbers of group targets.

Figure 8 shows the OSPA distance of the proposed algorithm and JPDA-based, PMHT-based, and PHD-based algorithms. It is observed that the JPDA-based algorithm has a larger OSPA distance, and the proposed algorithm presents a considerable performance with a lower OSPA error. In Figure 9, the average number estimation of group targets is apparent. This further highlights the drawback of the JPDA-based, PHD-based, and PMHT-based algorithms during group merging (at the 100 s time-point) and splitting (at the 125 s time-point), as JPDA-based and PHD-based algorithms lack a group structure model and the PMHT-based algorithm has flaws in its group partition vector. The superior performance of the proposed algorithm stems from the interaction among the group partition vectors and BP association optimization.

**Figure 10:**
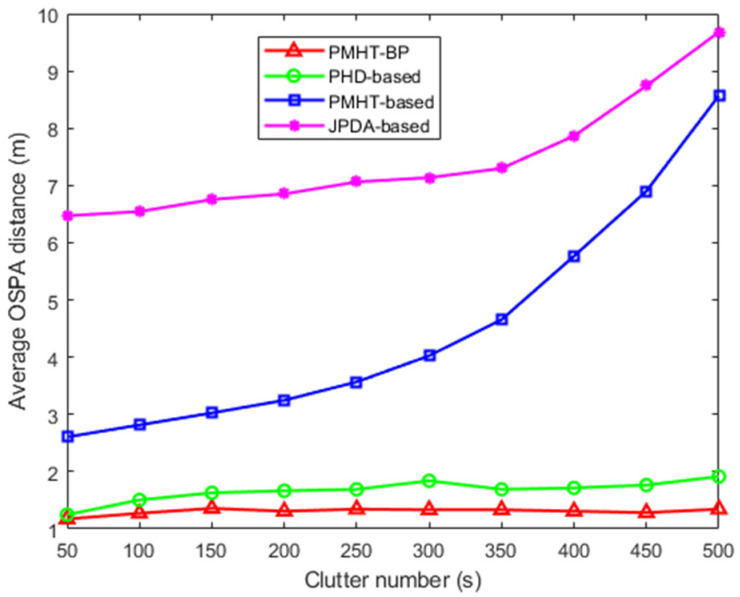
The average OSPA distances versus the clutter number.

To illustrate the influence of clutter parameters, the average OSPA distances with different clutter parameters are shown in Figure 10. From the figure, it is clear that the average OSPA distance of JPDA-based and PMHT-based algorithms increases rapidly with increased clutters parameters, and in comparison the proposed algorithm has better tracking accuracy and robustness.

## 5. Conclusions

In this paper, we have proposed a probabilistic multiple hypotheses tracking method based on belief propagation for multiple group targets with splitting–merging scenarios. First, we used the minimum spanning tree divisive clustering algorithm to classify the measurements and compute the expectation of the log-likelihood function of the measurements and association vectors. Subsequently, a data association factor graph model was constructed based on the group target measurement hypothesis. By using the belief propagation strategy, the joint posterior probability density of group target measurement associations and the marginal posterior association probability density were calculated. In the maximization step of the EM algorithm, the updated group target state was obtained by maximizing the group target state likelihood function. Simulation results demonstrate that the proposed PMHT-BP method outperforms the existing JPDA-based, PMHT-based and PHD-based methods. Future work includes applying the proposed algorithm to multi-sensor information fusion for multiple group target tracking in high-maneuverability scenarios.

## Figures and Tables

**Table 1:** Motion state of target parameters.

Group Target	Initial State (m)	Appearance Time (s)	Merger Time (s)	Split Time (s)	Disappearance Time (s)
1	[0, 5]	1	30	125	175
2	[0, 70]	1	30	125	175
3	[50, 5]	50	100	No split	175

## Data Availability

The original contributions presented in this study are included in the article. Further inquiries can be directed to the corresponding author.
